# Serological survey to determine measles and rubella immunity gaps across age and geographic locations in The Gambia: a study protocol

**DOI:** 10.1080/16549716.2025.2540135

**Published:** 2025-08-20

**Authors:** Oghenebrume Wariri, Abdul Khalie Muhammad, Alieu Sowe, Julia Strandmark, Chigozie Edson Utazi, C. Jessica E. Metcalf, Beate Kampmann

**Affiliations:** a Vaccines and Immunity Theme, Medical Research Council Unit The Gambia at London School of Hygiene and Tropical Medicine, Fajara, The Gambia; b Department of Infectious Disease Epidemiology, London School of Hygiene and Tropical Medicine, London, UK; c Data Science Cluster, Medical Research Council Unit The Gambia at London School of Hygiene and Tropical Medicine, Fajara, The Gambia; d Public Health, Ministry of Health and Social Welfare, Banjul, The Gambia; e WorldPop, School of Geography and Environmental Science, University of Southampton, Southampton, UK; f Southampton Statistical Sciences Research Institute, University of Southampton, Southampton, UK; g Department of Ecology & Evolutionary Biology, Princeton University, Princeton, NJ, USA; h Centre for Global Health, Charité Universitatsmedizin Berlin, Berlin, Germany

**Keywords:** Measles, rubella, immunity, serology, serosurvey

## Abstract

Vaccine coverage and disease surveillance data are valuable for monitoring protection against vaccine-preventable diseases; however, they do not directly measure population immunity. High-quality, representative serological studies can provide key insights into immunity gaps, outbreak susceptibility, and inform targeted vaccination strategies, even in high-performing immunization programs. This study aims to estimate location-specific and age-specific immunity profiles for measles and rubella while evaluating the predictive value of indirect immunity estimates derived from vaccination and surveillance data against direct serological measurements. Additionally, it seeks to model the risk of measles outbreaks and assess the impact of mitigation strategies. A multi-stage, stratified cluster sampling design will be implemented across six districts in The Gambia’s North Bank and Upper River Regions. Survey clusters (i.e. 5 km × 5 km areas) encompassing all settlements within their boundaries will be selected, proportionally to district population sizes. Cluster selection ensures representativeness of both the population and vaccine coverage within each district. Based on detecting a 10% difference in protective immunity across vaccine coverage levels, power analysis assumes an intraclass correlation coefficient (ICC) of 0.01. In each cluster, 70 children aged 9 months to 14 years will be recruited, yielding a total sample size of 1,750 children across 25 selected clusters. Dried blood samples will be collected and tested for anti-measles and anti-rubella IgG using enzyme immunoassays (EIA). District-specific measles seroprevalence will be estimated using a hierarchical spatial model. This study will generate key evidence needed to refine immunization strategies and reduce the risk of measles and rubella outbreaks.

## Paper Context

**Added knowledge:**

Representative measles and rubella serological studies can provide critical insights into immunity gaps, outbreak susceptibility, and targeted vaccination strategies, even in high-performing immunization programs.

**Global health impact on policy and action:**

This study will generate serological data to identify immunity gaps, refine immunization strategies, and strengthen measles and rubella outbreak prevention strategies in The Gambia.

## Background

Measles is highly contagious, requiring vaccination coverage rates and population immunity levels of 92–95% to prevent outbreaks or interrupt transmission [1]. However, despite high overall vaccination coverage rates, measles outbreaks can still occur due to factors such as waning immunity in certain age groups and untimely vaccination [2–4]. Thus, measles outbreaks serve as a sensitive indicator of emerging immunity gaps, even in populations with otherwise high vaccination coverage. The World Health Organization (WHO) African Region aimed to eliminate measles and rubella by 2020 [5,6]. However, transmission persists, with repeated measles outbreaks occurring even in countries that have achieved high vaccination coverage through routine and supplementary immunization activities (SIAs) [5]. While these outbreaks may be self-limiting, those who do become infected can suffer severe long-term complications, including blindness, deafness, and immune system impairment [7]. In the short term, immune system effects can persist for months, leaving children vulnerable to other serious infectious diseases [8].

Rubella infection, on the other hand, is typically asymptomatic or presents as a mild illness, which makes it difficult to detect and often leads to underreporting [7]. In African settings, the herd immunity threshold required to interrupt rubella transmission is estimated to be between 85% and 91% [9]. Failure to achieve this level of immunity in childhood increases the average age of infection, leaving women vulnerable to rubella infection during their reproductive years. This vulnerability heightens the risk of exposure and infection during pregnancy. Primary rubella infection during early pregnancy can have severe consequences, including miscarriage, stillbirth, or the birth of a child with congenital rubella syndrome (CRS), which carries serious public health implications [10]. The primary goal of rubella vaccination is to prevent CRS, with the WHO estimating that 100,000 CRS cases occur globally each year [11].

The Gambia achieved a global milestone in 1967 for being the first country to successfully interrupt measles transmission [12]. To sustain this achievement, the country launched its Expanded Programme on Immunization (EPI) in 1979, initially including the measles vaccine among seven core vaccines [13]. Routine first-dose measles vaccination (MCV1) was introduced at nine months of age [13], with a vaccine effectiveness (VE) of ~95% [14]. A second dose (MCV2), with a reported VE of ~99% [14], was implemented in 2012 [15], targeting children at 18 months. In 2017, the country switched from a measles-only vaccine to a combined measles/rubella vaccine (MR) for both MCV1 and MCV2 due to epidemiological evidence of rubella in the population. The Gambian EPI continues to be a model for sub-Saharan Africa, maintaining high routine vaccination coverage rates comparable to high-income countries. In the years leading up to the COVID-19 pandemic, the country consistently achieved MCV1 coverage rates exceeding 90%; however, MCV2 coverage consistently declined from a high of 81% in 2015, to 52% in 2022 [16]. Additionally, six SIAs for measles and rubella have been conducted since 2002 at intervals of 4–6 years, mostly targeting children aged 9 to 59 months. Coverage for all SIAs exceeded 91%, except in 2022, when it dropped to 53% [17]. This decline prompted a repeat SIA in 2023, which successfully reached a coverage rate of 88.4% [18].

**Figure 1.**
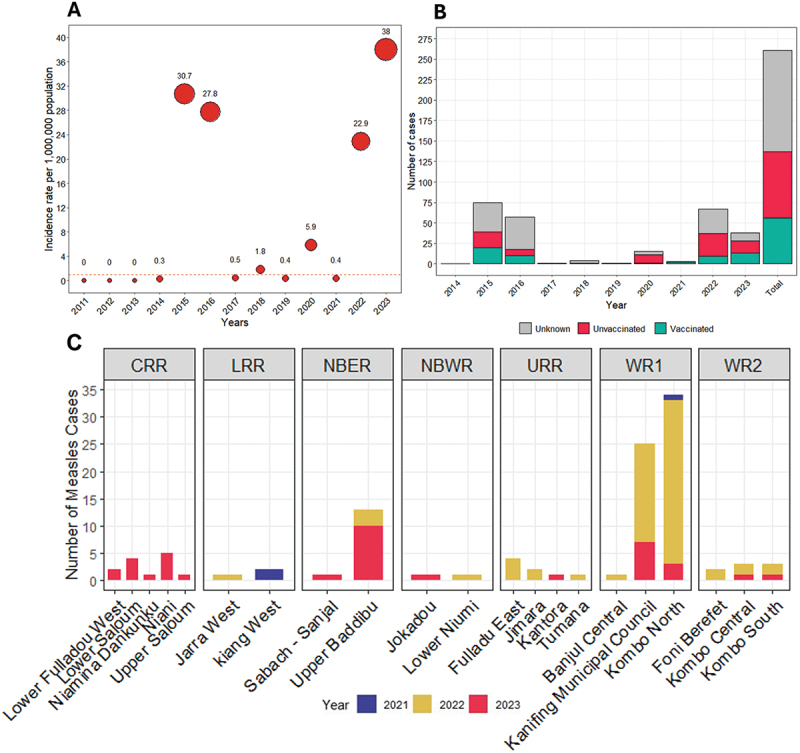
(A) Annual measles incidence rate per million population in the Gambia, 2011–2023; (B) Vaccination status of confirmed measles cases in the Gambia, 2014–2023; (C) Measles case distribution by district and year across the seven health regions in the Gambia. Note: The red horizontal dashed line is the WHO threshold for measles elimination of ≤ 1 case per million population. Data source: Gambia measles case-based surveillance database. CRR = Central River Region, LRR = Lower River Region, NBER = North Bank East Region, NBWR = North Bank West Region, URR = Upper River Region, WR1 = Western Region 1, WR = Western Region 2.

Recent measles outbreaks in The Gambia highlight the need for further investigation, especially considering the substantial progress the country had made toward the WHO measles and rubella elimination milestones by 2019 [19]. By mid-2023, there was a six-fold increase in measles cases compared to 2020 figures (Figure 1A) [20,21], despite historically high MCV1 coverage, the introduction of MCV2 for about a decade, and regular SIAs. Between 2014 and 2023, The Gambia reported 261 confirmed measles cases, with most cases occurring among children aged 1–4 years (35.2%), 5–9 years (33.0%), and under 1 year (12.3%) [20]. Additionally, 21% of all cases were reported among children who had received MCV1 (Figure 1B) and were mostly clustered in specific districts across the country (Figure 1C). Evidence suggests that the potential for measles outbreaks might be higher than expected in areas where there is geographic clustering of under-vaccinated children, even in settings with high overall measles vaccine coverage [22]. Previous studies from the US [2], Malawi [23], and The Gambia [3] have demonstrated that measles outbreaks can occur even with high overall vaccination coverage rates. This highlights the importance of thoroughly mapping population immunity profiles and predicting outbreak susceptibility across different locations and age groups in the country.

A key challenge in understanding infectious disease dynamics is accurately defining the landscape of population immunity and susceptibility to outbreaks [14,24]. Although vaccine coverage and disease surveillance data are often used to monitor population immunity levels and identify susceptible subpopulations, these data sources do not directly measure immunity across age and space [14,25]. Issues such as overestimated vaccination coverage rates from administrative or survey-based data, along with under-reported cases due to poor surveillance systems, can lead to an overestimation of immunity [14,26]. This can result in masking of immunity gaps and clustering of outbreak risk within specific subpopulations – a problem observed in many low-income countries, including The Gambia.

High-quality, representative and geographically diverse serological surveys provide the most direct and accurate method for defining the immunity landscape [27]. Serological surveys are useful in predicting outbreak susceptibility, and informing targeted vaccination strategies, particularly when longstanding immunity gaps are suspected in specific age groups or sub-populations [26]. These surveys can help resolve uncertainties and identify when, where, and in whom immunity gaps are significant. Despite their value, serological surveys are underutilized due to logistical challenges [28], lengthy implementation times, and the lack of reliable, representative sampling frames [14]. Additionally, obtaining representative samples can be difficult, and it is rare to have access to critical metadata, such as immunization records, alongside available samples [29]. Cost concerns also make serosurveys less feasible in low- and middle-income countries (LMICs) [24], where they have been conducted less frequently [30]. The WHO recommends less invasive methods, like dried blood spot collection, for large surveys as these simplify specimen collection, lower costs, and improve participation [14]. However, venous blood collection, the gold standard, is more invasive and may reduce survey uptake. Our unique access to the health and demographic surveillance systems (HDSSs) in The Gambia provides an opportunity to overcome some of these challenges.

## Methods

### Study aim and objectives

The main aim of this study is to investigate the population-level measles and rubella immunity gaps across age and spatial location across two regions in The Gambia. The findings will help predict outbreak susceptibility and inform targeted vaccination strategies aimed at measles and rubella elimination in the country.

The primary objectives are;

1. To estimate the age-specific and location-specific immunity profiles for measles and rubella across two regions in The Gambia.
2. To evaluate the predictive value of indirect estimates of measles and rubella population immunity (based on vaccination and surveillance data) compared to direct immunity measured through cross-sectional serological data.
3. To model the risk of a measles outbreak across different age cohorts and spatial locations in The Gambia based on seroprevalence data.

The secondary objectives are;

1. To assess the impact of mitigation strategies, such as adjustments to routine vaccination and supplementary immunization activities, on the modeled risk of a measles outbreaks.
2. To compare seroprevalence levels determined using dried blood spots versus venous blood specimens for estimating measles and rubella seroprotection in The Gambia.

### Study design

This study is a cross-sectional serological survey to estimate measles and rubella immunity gaps across age and spatial location in The Gambia.

### Study setting and population

**Figure 2.**
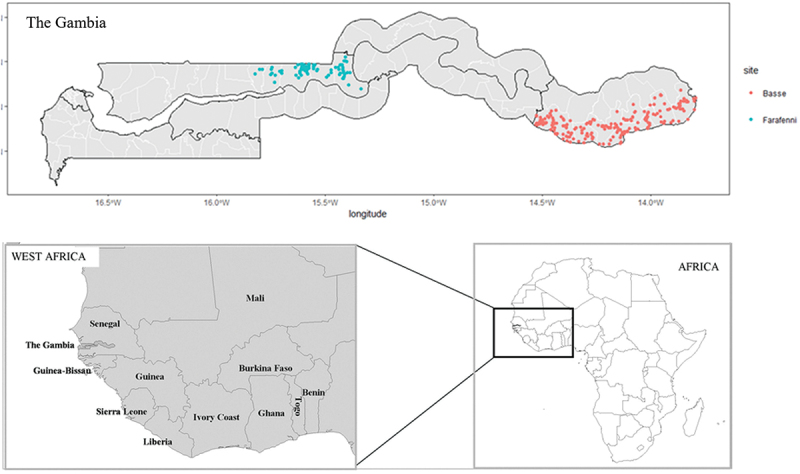
Spatial locations of villages/settlements in the Basse and Farafenni Health and demographic surveillance systems (HDSS) located in the Upper River and North Bank Region of the Gambia.

Note: Each blue dot in The Gambia map represents villages or settlements included in the Farafenni HDSS, and each red dot represents those in the Basse HDSS.

The study will be conducted within the Basse and Farafenni Health and Demographic Surveillance Systems (BHDSS and FHDSS), located in the Upper River and North Bank Regions (URR and NBR) of The Gambia, respectively (Figure 2). The BHDSS and FHDSS collectively monitor approximately 250,000 individuals, representing about 10% of the country’s population [31]. With a combined annual birth cohort of about 8,000 infants, both HDSS sites conduct routine surveillance rounds every four months, collecting comprehensive health information, including vaccination and disease data from all consenting households within the communities.

The geographic location and population covered by both HDSS sites present a unique opportunity to gain insights into the dynamics of measles and rubella. Notably, disease surveillance data show that NBR, home to the FHDSS, reported the second-highest number of measles cases in 2022, surpassed only by the more urban Western Region. By 2023, NBR recorded the highest number of measles cases in The Gambia (Figure 1C). This highlights the critical need to implement serosurveillance in this part of The Gambia to better understand immunity gaps across different age groups and at finer spatial scale. Additionally, our previous research has identified URR, where the BHDSS is situated, as one of the two regions with the highest rates of delayed MCV1 [32], further justifying the implementation of serosurveys in these areas.

For this serological survey, we will recruit children aged nine months to 14 years, categorized into four age cohorts: < 1 year, 1–4 years, 5–9 years, and 10–14 years. This stratification aligns with disease surveillance data indicating higher measles prevalence within this age range in The Gambia [20]. It enables a detailed analysis of serological profiles across age groups, providing key insights for The Gambia immunisation program to inform targeted vaccination strategies.

### Sampling strategy and sample size

#### Methodological overview

We integrated geospatial data and demographic projections (i.e. population and age categories) to establish a robust sampling frame for children aged 9 months to 14 years in the two HDSS sites (i.e. the BHDSS and FHDSS). Our approach combines:

- Choropleth Map: Derived from the 2019–2020 Gambia Demographic and Health Survey (GDHS), this map models measles and rubella first-dose (MR1) vaccine coverage at 9 months across 5 km × 5 km raster grids for children aged 12–35 months. Each raster grid, assigned a uniform MR1 coverage, represents all settlements within that grid cell.
- Point Map: Obtained from the BHDSS and FHDSS, this map provides the geographical coordinates (longitude and latitude) of all settlements in BHDSS and FHDSS. The point map showing the settlements was overlaid on the choropleth raster map.

#### Population projection

We extracted population data from BHDSS and FHDSS as of January 2023:

- BHDSS: Comprises 220 villages with 43,428 children 9 years and under (3,223 aged 0–1 years 25,608 aged 1–4 years, and 14,597 aged 5–9 years).
- FHDSS: includes 149 villages with 13,858 children 9 years and under (944 aged 0–1 year, 8,111 aged 1–4 years, and 4,803 aged 5–9 years).

Children aged 10–14 years will also be included in the serosurvey. Since data collection is scheduled for April – August 2025, we projected population sizes for each settlement using time series forecasting (*auto.ARIMA* models) applied to monthly HDSS birth data available through January 2023. These projections account for children aged 9 months to 14 years, ensuring that the sampling frame reflects anticipated demographic changes.

#### Sampling strategy

A multi-stage stratified cluster sampling design was employed across BHDSS and FHDSS. The sampling procedure occurred in four stages: stratification by site and district, selection of clusters, selection of households, and selection of children.

##### Stage 1: stratification by site and district

The study population is first stratified by HDSS site (i.e. BHDSS and FHDSS) and further by district within each site. There are a total of six districts: four in BHDSS and two in FHDSS.

##### Stage 2: selection of clusters

Survey clusters were defined as 5 km by 5 km land areas (25 km^2^), corresponding to the raster resolution of the modeled MR1 coverage from the 2019–2020 GDHS [33]. The primary sampling units (PSUs) are these clusters and includes all HDSS settlements within its boundaries. Clusters were selected within each district proportional to the district’s population size, ensuring that districts with larger populations contribute more clusters to the sample. Additionally, the selection process ensured that the combination of clusters were representative of both the population distribution and the overall MR1 coverage within each district.

For each cluster, let: ***a*** = the population size of the cluster; *b* = the cumulative population size of all clusters within a stratum (district); *c* = the predetermined number of individuals to be sampled from the cluster; and *d* = the total number of clusters to be sampled within the stratum (district).

The sampling interval (SI) per district is calculated as:

$$\mathrm{SI}=\frac{b}{d}$$

To ensure randomness across districts, a random start (RS) between 1 and SI is chosen, and clusters are selected according to the arithmetic progression:

$$n_{\mathrm{th}}\mathrm{value}=\mathrm{RS}+\left(n-1\right)\times \mathrm{SI}$$

using the cumulative population sizes to determine the sequence.

To adjust for potential sampling biases and to ensure that the sample is representative of the population, inverse probability weighting (IPW) was applied. The probability of selecting a cluster *(P*_*clust*_) and an individual within the cluster *(P*_*indiv*_) are computed as:

$P_{\mathrm{clust}}=\frac{a\times d}{b}$ and $P_{\mathrm{indiv}}=\frac{c}{a}$

Thus, the IPW for each individual is:

$$\mathrm{IPW}=\frac{1}{P_{\mathrm{clust}}\times P_{\mathrm{indiv}}}$$

Clusters with fewer than 40 children aged 14 years or younger were excluded from the sampling frame to avoid selection bias and ensure statistical robustness. This threshold ensures that each selected cluster contributes sufficient data for meaningful analysis, particularly for the 1–4 years age group, which the study is powered by and is critical for assessing vaccine coverage and seroconversion following earlier MR1 and MR2 vaccination.

**Figure 3.**
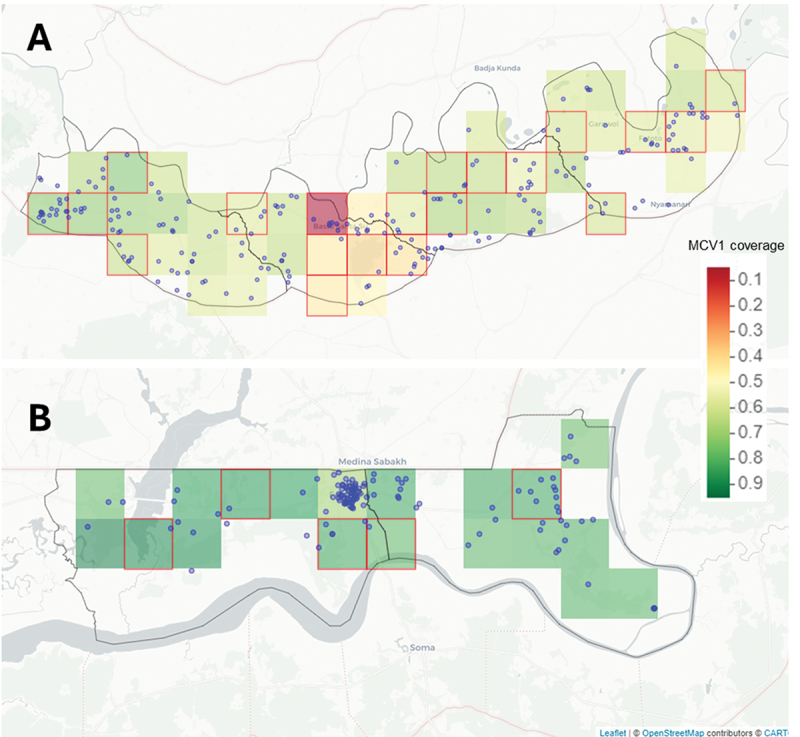
The distribution of randomly selected serosurvey clusters across the (A) four districts in the Basse HDSS area and (B) two districts in the Farafenni HDSS area. Note: The green, yellow, and red tiles represent MCV1 coverage data at a 5 km by 5 km resolution based on The Gambia DHS (2019–2020) [33]. Blue dots indicate settlements/villages within the Basse and Farafenni HDSS sites, and red squares indicate the 25 selected 5 km by 5 km clusters.

Following cluster selection, a statistical power analysis was conducted. This analysis, based on detecting a 10% difference in protective immunity between different MR1 coverage levels and assuming an intraclass correlation coefficient (ICC) of 0.01 was conducted using simulations on a high-performance computing (HPC) system. The sampling procedure was simulated 10,000 times, and the final sample consists of the top 5% of simulations with the highest statistical power (see supplementary Table S1). Figure 3 shows the map of the 25 selected clusters from the BHDSS and FHDSS.

##### Stage 3: selection of households

Within each selected cluster, a comprehensive list of households – defined according to the HDSS and spanning all settlements – will be extracted from the HDSS database. To ensure representativeness, this list will be randomized, and the first 40 households will be selected for inclusion. In clusters with fewer than 40 households across all the settlements, all households will be included. If the initially selected 40 households yield fewer than the expected number of eligible children, additional households from the randomly generated list will be sampled until the target is reached. Selected households will then be assigned to data collectors for field visits.

##### Stage 4: selection of children

From the selected households, a list of all eligible children will be made. To ensure adequate power and based on the simulations conducted, 70 children per cluster will be sampled from these households according to the following age group allocation: < 1 year (10 children); 1–4 years (40 children); 5–9 years (10 children); 10–14 years (10 children).

This allocation (ratio 1:4:1:1) was chosen because the study was powered based on the 1–4 years age group, which is key to assessing vaccine coverage and immunity profiles following MR1 and MR2. In households containing multiple children within the same age cohort, one child per cohort will be randomly selected, ensuring that no more than one child from each age group is chosen from the same household. Sampling in each cluster will continue until 70 children are recruited, yielding a total sample size of 1,750 children from 25 selected clusters. To avoid selection bias, the survey Statistician and Data Manager, instead of field teams, will be responsible for the selection of households and generating random list of children to be recruited from each cluster.

### Community and individual sensitization

To ensure ethical and comprehensive engagement, we will conduct both community and individual sensitization activities before obtaining informed consent and initiating any study procedures or data collection.

#### Community sensitization

Community sensitization will involve consultation with the Ministry of Health (both national and regional health directorates) and key stakeholders, including EPI program managers, representatives from WHO in The Gambia and district leadership. Before commencing study activities, we will organize a series of *kolanut* meetings with prominent leaders in the selected local communities [34]. These leaders include the District Chiefs, *Alkalos* (village/community leaders), other community elders, religious leaders, women’s and mothers’ group representatives, and other local advocates. The meetings will be held at accessible venues within the chosen survey communities.

During these meetings, we will discuss the details of the serosurvey, allowing ample time for questions to ensure that everyone present fully understands the purpose of the survey, as well as other key information, such as potential risks and benefits. The information provided will be based on the informed consent document (ICD) to ensure accuracy and consistency.

Following the meetings, we will disseminate information about the serosurvey through established community networks to reach a broader audience [34]. These engagements aim to foster understanding among influential community members, minimize misconceptions, and encourage accurate communication within the community. Feedback received during these sessions will guide the planning of individual sensitization activities.

#### Individual sensitization

After the community sensitization, we will conduct individual sensitization to provide more detailed information about the serosurvey directly to the parents or caregivers of children within the eligible age cohorts. During these visits, which will take place at the homes of the parents or caregivers of the selected children, the study team members will summarize the key information from the approved ICD.

Parents or caregivers will receive a copy of the ICD and be encouraged to review it with their partner and other significant family members. During this visit, we will also collect basic contact information, such as telephone numbers, to facilitate follow-up communication. A subsequent visit, scheduled at least 24 hours later, will provide an opportunity to address any additional questions and obtain formal informed consent. Only after obtaining this consent will any study procedures or data collection activities be initiated.

### Informed consent procedure

Before any study procedures or data collection take place, signed or thumb-printed, dated, and timed written informed consent and assent for participation in the serosurvey must be obtained using an ICD approved by the Gambia Government/MRC Joint Ethics Committee and the LSHTM Research Ethics Committee. The identity and age of all prospective participants will be confirmed using a suitable source document, such as a birth certificate, or the hand-held infant welfare card.

Written informed consent will be sought from all parents of the selected children within the eligible age cohorts. For children aged 6–11 years, we will also confirm their willingness to participate and document their verbal assent, while for those aged 12–14 years, we will seek written assent in addition to parental written informed consent. Study staff (nurses and field workers) with appropriate English and local language skills will obtain informed consent and assent. Consent can only be obtained from parents who are at least 18 years old. Therefore, if both parents of a child are under 18, the child cannot be enrolled in the study. Whether the process is conducted in English or a local language, the ICD will be reviewed line-by-line with the parent or caregiver by the staff conducting the consent process to ensure all details are understood. Self-reading alone will not suffice. During this review, opportunities for questions and clarifications will be provided. Since the local languages in The Gambia are primarily spoken and not widely written, the ICD will be provided in English and translated orally into the relevant local languages by the person obtaining consent.

Consent from a guardian, rather than a parent, will only be accepted if both parents are deceased or if both parents are out of The Gambia for an extended period, leaving another adult to care for the child. Guardians cannot provide consent if the parent is in another part of The Gambia or temporarily out of the country. A certified copy of the completed ICD will be provided to the parent or caregiver after the consent is signed or thumb-printed. The outcome of the informed consent process (i.e. consented/did not consent) will be recorded in the study’s consent log. As part of the informed consent process, consent will also be sought for storage of samples, the future use of any remaining samples and associated data for ethically approved research intended to benefit the people of The Gambia.

### Specimen and data collection

Specimen and data collection will be led by study nurses, who will be responsible for ensuring accuracy and completeness of information [34]. Sociodemographic data, vaccination history (campaign-based and routine vaccination), and any history of measles in the index child in the preceding one year will be collected from caregiver or parents and recorded using electronic Case Report Forms (eCRF). Hand held vaccination cards will serve as the primary sources of vaccination data, however, caregiver or parental recall will also be used when cards are unavailable. In addition, routinely collected vaccination records from the HDSS database will be used to supplement the data when needed.

Dried blood spots (DBS) will be collected from all children enrolled in the study and will occur in the participants’ home. To obtain the DBS, the fingertip of each child will be wiped clean with alcohol swabs and pricked using a contact-activated retractable lancet. Two to three blood droplets (approximately 150 μl of whole blood) [35] will be collected directly onto labeled *HemaSpot HF* device (SpotOnSciences, San Francisco, CA, USA) within 30 minutes of opening the device. The *HemaSpot HF* cartridge contains an absorbent collection matrix with a desiccant to protect the sample from moisture and the collection component is covered by an application surface with a small opening to allow biological fluids to enter. Once two to three drops of blood are collected, the cartridge will be closed within one minute, to allow the built-in desiccant to dry the sample within minutes. For DBS stability in our setting with high humidity, cartridges will be transported at ambient temperature from the field but stored at 4°C for longer periods in the lab [36]. All samples will be collected following universal precautions.

To compare the diagnostic accuracy of DBS with venous blood samples for estimating measles and rubella population immunity levels, an additional venous blood sample will be collected concurrently with the DBS from a randomly selected 10% of the study participants. Children will be eligible for venous blood collection if they are at least one-year-old, reside in a cluster with a health facility, and where samples can be transported to the laboratory within 30 minutes. Venous blood collection will take place in a health facility or similar setting within the selected clusters. For these participants, up to 2 ml of venous blood will be drawn into a serum separator tube, immediately following the collection of the DBS sample. Serum tubes will be gently inverted 5 times and the blood left to stand upright for 30 minutes at ambient temperature to allow clotting. Samples will then be stored at 4 to 8°C in cold boxes and transported to the laboratory. In the laboratory, specimens will be recentrifuged at 1000 g for 10 minutes and the sera aliquoted, labeled, and stored at − 20°C.

If a child or parent refuses venous blood collection, they will be replaced by another child from the same age cohort and cluster. However, only the DBS sample will be collected from the original child who declined the venous blood draw.

### Sample testing

Measles and rubella virus-specific immunoglobulin G (IgG) antibodies are well-established markers of immunity against infection and of vaccination. Paired DBS and serum samples will be tested on the same plate using commercially available anti-measles IgG and anti-rubella IgG enzyme immunoassay (EIA) kits (Euroimmun, Perkin Elmer, Germany) [36,37]. Serum samples will be processed according to the manufacturer’s protocol, with samples diluted 1:101 using the Euroimmun ELISA kit sample buffer prior to testing. DBS samples will first be eluted following a method described elsewhere [36]. Briefly, one wedge will be selected from each *HemaSpot HF* device and placed into a cryotube containing 100 μl of elution buffer. Tubes will be incubated for 2 hours at 37°C, with agitation for 20 minutes before and after incubation after which filter paper will be discarded and samples centrifuged followed by collection of eluate.

Based on extensive standardization testing for optimal elution and dilution ratios, 30 μl of eluted DBS will be diluted using 210 μl of the Euroimmun ELISA kit diluent, separately for both measles and rubella, at a 1:8 ratio for the DBS samples [36]. All subsequent steps for ELISA testing will be identical for sera and DBS. Seroprotection levels will be interpreted quantitatively and qualitatively based on the manufacturer’s recommendations. Near-real-time data management will be conducted to monitor variability. For quality assurance, a randomly selected stratified subset of serological samples, including all those with equivocal results, will be re-tested. If a specimen result remains equivocal after re-testing, it will be classified as negative to ensure consistency and to err on the side of slightly underestimating the population-level immunity prevalence. We plan to conduct all tests at the MRC Unit The Gambia serology laboratories. However, if this is not possible for any reason, we may ship the samples to another laboratory outside The Gambia for testing.

### Data management

An approved data management plan has been prepared and is available upon request. Data collection will be conducted using eCRFs in a standardized format implemented on the Research Electronic Data Capture (REDCap) platform. Electronic data capture will occur offline using encrypted devices, which will be synchronized with the central online server on a daily basis.

REDCap incorporates front-end data quality checks to ensure accuracy during data entry. In addition, back-end edit and validation checks are embedded to monitor data validity. These checks include identifying inconsistent dates and times, as well as entries that fall outside predefined ranges. Any data queries identified during these checks will be generated weekly by the data manager and resolved promptly by the study field team. Periodic reports on data quality will be produced to ensure continuous monitoring.

All data will be stored securely on access-controlled computers and servers in compliance with MRC Unit The Gambia’s (MRCG) data management policies. The study database will undergo regular backups as part of the MRCG’s IT disaster recovery policy [34]. Any paper records (e.g. consent forms) will be retained for a minimum of 10 years in the Unit’s archiving facility. Data and biological samples will be stored in a linked-anonymized format, using participants’ study numbers. No identifiable participant information, such as names or addresses, will be stored in the eCRF or linked to study samples. This approach ensures the confidentiality, integrity, and security of all data and aligns with best practices in data management.

### Statistical analysis and modeling

#### Descriptive analysis

We will use the R package *survey* to account for the complex sampling design when estimating age-specific and location-specific measles and rubella seroprevalence. Sampling weights will be applied to ensure representativeness, given the probability proportional to size sampling across the 25 randomly selected clusters. The weights will be computed based on the inverse probability of selection and will be adjusted to account for differences between the projected and actual population sampled per age group in each cluster.

Seroprevalence estimates will be generated for the four age cohorts (under 1 year, 1–4 years, 5–9 years, and 10–14 years) and across the sampled districts. We will report overall, HDSS level, district, ward, and age-specific seropositivity estimates. All 95% confidence intervals will be computed using linearized Taylor series variance estimation. We will use T-tests to assess the significance of differences in weighted seroprevalence between subgroups (e.g. age, sex, district). Associations between seropositivity and predictor variables (age, sex, geographic location) will be analyzed using univariate and multivariate generalized linear regression models, adjusting for the survey weights. Both unadjusted and adjusted odds ratios (ORs) with 95% confidence intervals will be reported.

#### Main analysis

To model age-related seroprevalence for measles and rubella, we will employ hierarchical generalized additive models (GAMs) fitted to individual-level seropositivity data. These models will include HDSS area-level smoothers over age. Model selection will be based on the Akaike Information Criterion (AIC) for optimal fit.

For district- and ward-specific (i.e. the admin level below the district level) spatial seroprevalence estimates, continuous geospatial models will be fitted, under the assumption that neighbouring districts and wards share more similar seroprevalence patterns than distant ones. Model covariates may include district-level routine vaccination coverage, population density, and other demographic and geospatial factors. The final spatial models will be selected based on the Widely Applicable Information Criterion (WAIC) or using cross-validation approaches.

A comparison of indirect immunity estimates, derived from vaccination coverage and surveillance data, with direct serological data will be evaluated using receiver operating characteristic (ROC) curve analysis. Sensitivity, specificity, and area under the ROC curve (AUC) will be computed to measure the agreement between indirect estimates and the actual serological findings. We will also model the risk of a measles outbreak across different age groups and geographic clusters. Using the basic reproductive number (R0) for measles, we will estimate the proportion of the population susceptible to infection.

Spatial epidemiological models, specifically Bayesian hierarchical models, will account for geographic heterogeneity and clustering of immunity gaps. We will use the *spdep* and *INLA* R packages for spatial modeling. Simulation models will evaluate the impact of various vaccination strategies on outbreak risk, incorporating seroprevalence estimates and vaccination coverage data. Scenarios will assess the impact of adjustments to routine immunization schedules and SIAs on outbreak risk.

All analyses will be performed using R (version 4.0.5). Key R packages include *survey*, *mgcv* (for GAMs), and *INLA* (for spatial models). Supplementary analyses will be conducted as necessary.

### Dissemination plans

We will publish the results of the serosurvey in open-access, peer-reviewed journals and presented at relevant global health conferences and to key stakeholders. Additionally, we will share the findings with the study communities during *Open Days* conducted at the end of the study. Given the importance of these results for the measles and rubella elimination strategy in The Gambia, we will also disseminate the findings to the EPI program and relevant partners.

### Ethics

The Gambia Government/MRCG Joint Ethics Committee granted ethical approval for this study (Project ID/Ethics ref.: 31364; Date: 6 December 2024). The London School of Hygiene and Tropical Medicine Research Ethics Committee also granted ethical approval for this study (LSHTM Ethics Ref: 31364, 20 January 2025).

## Discussion

The findings from this study will provide critical insights to guide The Gambia EPI and similar programs in LMICs. Despite The Gambia’s reputation as a model immunization program in sub-Saharan Africa, achieving routine vaccination coverage levels comparable to many high-income countries, measles outbreaks persist at rates exceeding the WHO elimination threshold of one case per million population [38]. Additionally, rubella surveillance remains suboptimal in The Gambia and many LMICs, limiting the ability to track immunity gaps. By estimating age-specific and location-specific immunity profiles, this study will help identify populations at risk of measles outbreaks and CRS, allowing immunization programs to tailor interventions more effectively. A key strength of the study is its evaluation of indirect immunity estimates derived from vaccination and disease surveillance data against direct measles and rubella serological measurements. Given that serological surveys are logistically challenging and costly, this analysis could improve the ability to predict herd immunity levels in the absence of serological data. Ultimately, the study’s findings will refine immunization strategies, address immunity gaps, and contribute to reducing the risk of measles and rubella in The Gambia, thereby strengthening public health outcomes.

Methodologically, this study has several strengths that enhance the reliability of its findings and applicability in other settings. Unlike previous retrospective studies that relied on banked serological samples [30,39,40], which may not be representative of the broader population, this prospective study employs a multi-stage stratified cluster sampling design, ensuring a representative and robust dataset. This approach allows for extrapolation of findings to the study population with greater confidence. Additionally, embedding the study within two well-established HDSS areas provides access to detailed demographic and vaccination data, ensuring the appropriate population is included and enhancing the accuracy of the immunity estimates. Critical metadata, such as dates and doses of routine and campaign-based measles and rubella vaccinations, as well as reported measles cases, will be incorporated into the analytic models, strengthening their predictive power. Furthermore, by including two geographically diverse locations; NBR, where measles vaccine coverage is higher, and URR, where vaccine coverage is lower with higher rates of delayed vaccination, the study potentially captures a broad spectrum of immunity patterns. This geographic diversity allows for a nuanced understanding of how vaccination coverage and timeliness influence immunity levels, ultimately improving the ability to design targeted immunization strategies.

## Supplementary Material

Supplementary_material_FINAL_ZGHA_2025_0063.R1.docx

## Data Availability

Data sharing is not applicable to this article as no data were created or analyzed in this study.
