# Supplementary material for: Serological survey to determine measles and rubella immunity gaps across age and geographic locations in The Gambia: a study protocol
Source: Glob Health Action. 2025 Aug 20;18(1):2540135. doi: 10.1080/16549716.2025.2540135 (PMC12372506; doi:10.1080/16549716.2025.2540135)
Supplement: Supplementary_material_FINAL_ZGHA_2025_0063.R1.docx [file ZGHA_A_2540135_SM6692.docx]

# Detailed information about Population Projection

As of January 2023, the Basse HDSS had 43,428 children under 9 years old: 3,223 were aged 0–1 years, 25,608 were aged 1–4 years, and 14,597 were aged 5–9 years. In the Farafenni HDSS, there were 13,858 children under 9 years old: 944 were aged 0–1 years, 8,111 were aged 1–4 years, and 4,803 were aged 5–9 years. In addition to these age groups, we will also include children aged 10 to 14 years in our sampling.

Since the survey will be conducted between April to August 2025, we projected the population sizes for each settlement to account for potential growth in the target age groups. Using available monthly HDSS birth data up to January 2023 and time series forecasting (*auto.arima models*), we estimated the population sizes for children aged 9 months to 15 years. The projections were based on settlement-specific *auto.arima* models, with a maximum configuration of (p,q,d,P,Q,D) set at (3,3,3,3,3,3). For likelihood estimation, we used the *Rossignol2011* method for state-space initialization, and the *BFGS algorithm* for optimization, allowing up to 300 models in the stepwise search. A single random value from the confidence interval (CI) for each projected month was used to estimate the population. In small settlements where the lower CI fell below zero, population estimates were set to zero for sampling purposes.

**Table S1**: Statistical power analysis

| **Districts** | **HDSS Site** | **No. Clusters Selected** | **Median MR1 Coverage** | **Power** |
| --- | --- | --- | --- | --- |
| Basse | Basse | 6 | 0.4233 | 0.7489 |
| Illiasa | Farafenni | 3 | 0.8724 | 0.9999 |
| Jimara | Base | 4 | 0.7302 | 0.8153 |
| Kantora | Basse | 5 | 0.6019 | 0.7461 |
| Sabach | Farafenni | 2 | 0.8212 | 0.8039 |
| Tumana | Basse | 5 | 0.6406 | 0.7814 |

Note: MR1 = 1^st^ dose of measles and rubella vaccine. The median MR1 coverage values reported are based on the modelled vaccination coverage raster surface at 5km by 5km from the 2019-2020 Gambia DHS for children 12-35 months.^1^ The reported power is based on detecting a 10% difference in seroconversion rates across measles vaccine coverage levels and assumed an intraclass correlation coefficient (ICC) of 0.01.

# References

1. Gambia Bureau of Statistics (GBoS) and ICF. The Gambia Demographic and Health Survey 2019-20. Banjul, The Gambia and Rockville, Maryland, USA, 2021.Reference
